# Isolation and Culture of Pig Spermatogonial Stem Cells and Their *in Vitro* Differentiation into Neuron-Like Cells and Adipocytes

**DOI:** 10.3390/ijms161125958

**Published:** 2015-11-04

**Authors:** Xiaoyan Wang, Tingfeng Chen, Yani Zhang, Bichun Li, Qi Xu, Chengyi Song

**Affiliations:** College of Animal Science & Technology, Yangzhou University, Yangzhou 225009, China; wxyan@yzu.edu.cn (X.W.); ctf20070702664@126.com (T.C.); ynzhang@yzu.edu.cn (Y.Z.); xuqi@yzu.edu.cn (Q.X.); cysong@yzu.edu.cn (C.S.)

**Keywords:** SSCs, *in vitro* culture, induction, neuron-like cells, adipocytes

## Abstract

Spermatogonial stem cells (SSCs) renew themselves throughout the life of an organism and also differentiate into sperm in the adult. They are multipopent and therefore, can be induced to differentiate into many cells types *in vitro.* SSCs from pigs, considered an ideal animal model, are used in studies of male infertility, regenerative medicine, and preparation of transgenic animals. Here, we report on a culture system for porcine SSCs and the differentiation of these cells into neuron-like cells and adipocytes. SSCs and Sertoli cells were isolated from neonatal piglet testis by differential adhesion and SSCs were cultured on a feeder layer of Sertoli cells. Third-generation SSCs were induced to differentiate into neuron-like cells by addition of retinoic acid, β-mercaptoethanol, and 3-isobutyl-1-methylxanthine (IBMX) to the induction media and into adipocytes by the addition of hexadecadrol, insulin, and IBMX to the induction media. The differentiated cells were characterized by biochemical staining, qRT-PCR, and immunocytochemistry. The cells were positive for SSC markers, including alkaline phosphatase and SSC-specific genes, consistent with the cells being undifferentiated. The isolated SSCs survived on the Sertoli cells for 15 generations. Karyotyping confirmed that the chromosomal number of the SSCs were normal for pig (2*n* = 38, *n* = 19). Pig SSCs were successfully induced into neuron-like cells eight days after induction and into adipocytes 22 days after induction as determined by biochemical and immunocytochemical staining. qPCR results also support this conclusion. The nervous tissue markers genes, *Nestin* and β*-tubulin*, were expressed in the neuron-like cells and the adipocyte marker genes, *PPAR*γ and *C/EBP*α, were expressed in the adipocytes.

## 1. Introduction

Spermatogonial stem cells (SSCs) are a unique population of cells in male testes. They have a dual role in both self-renewing their population to maintain stem cell pool and differentiating into spermatids in mammalian testis.After birth, SSCs, which are generally considered as type A spermatogonia in testis, will divide into B spermatogonia through mitosis. B spermatogonia become spermatocytes that enter meiosis to produce haploid spermatids. As such, they are an important current focus in studies of male infertility [1,2,3]. Spermatogonial stem cells are estimated to comprise only 0.03%–0.05% of germs cells in adult mice testis [4]. Their rarity has presented problems in isolation and culturing them and the development of successful techniques for doing so has been the focus of intense study. At present, SSCs have been successfully isolated and cultured from many species, including human, mice [5], cattle [6], pig [7,8,9], goat [10,11,12], dog [13], chicken [14,15], and even fish [16].

Induced pluripotent stem cells (iPSCs) are a type of stem cell that can be generated from adult cells. The first induction of SSCs to iPSCs was in mice and was achieved by Kanatsu-Shinohara (2004) [17]. Using genetic selection, Guan subsequently isolated adult mouse SSCs that possessed embryonic stem cell (ESC) properties and were capable of developing into various organs when injected into a blastocyst in an early developmental stage [18]. This method was improved upon by Ko [19]. Conrad (2008) successfully obtained human adult germline stem cells from adult human testis. These cells produced teratomas and, when grown under conditions that induce differentiation of human ESCs, differentiated into several different types of somatic cells, including types that are normally derived from all three germ layers [20].

There are numerous similar reports of differentiation of SSCs into a variety of somatic cell types, summarized below. Embryonic stem cell-like cells derived from adult human testis were induced into neurocytes, epithelial cells, osteoblasts, myogenic cells, adipocytes, and pancreatic cells [21]. When bovine SSCs were cultured with Sertoli cells in a consecutive inducer media, they differentiated into osteoblasts [22]. A spermatogonial cell line of medaka, the Japanese rice fish, was capable of differentiation into ectodermal and sodermal cells, adipocytes, melanocytes, neuron-like cells, and matrix-depositing osteoblasts [23]. Finally, we have successfully developed methods to differentiate chicken SSCs into osteoblasts, neuron-like cells, and adipocytes [24].

Expression of *Nanog* and *c-myc*, genes that code for transcription factors important in the self-renewal of undifferentiated stem cells and cell cycling, respectively, was elevated in ESC-like cells derived from neonatal mouse testis compared to the cells of origin. In contrast, the expression of *Stra8*, *mvh*, and *piwill2*, expressed only in spermatogonia, was less in these cells than in the control [25]. Finally, Asadi recently acquired ESC-like cells from neonatal mouse testis using an autologous Sertoli cells co-culture system and showed that expression of genes associated with pluripotency increased and expression of germ-cell specific genes decreased compared to the testis cells [26]. In total, this research confirms that SSCs can be reprogrammed to a pluripotent state. 

The recent interest and research on SSCs has led to the recognition that they have potential value in regenerative medicine [27,28]. Embryonic stem cells were considered to hold great promise in animal reproduction and research, but the moral, legal, and ethical considerations forbid their use in regenerative medicine. Induced pluripotent cells that are derived from somatic cells and can be genetically manipulated by transcription factors, such as OCT4 (a member of POU transcription factor family) or SOX2 (Sex determining region Y-box 2), are also considered promising. However, these have also been problematic as the exogenous genes could result in an increased risk of tumorigenicity [29]. If SSCs can be used as pluripotent cells source, problems associated with ESC-like cells or iPSCs can be avoided [30]. Stem cells or iPSCs are now widely used for repairing human tissues, mainly the nervous system [31,32,33]. SSCs hold great potential for use in clinical treatments.

Here, we report on our continued efforts to establish an *in vitro* culture system for SSCs. We have chosen to conduct this study with pig tissues because there are considerable similarities between the anatomy and physiology of pigs and humans. Also, pigs are free from the ethical issues associated with human tissues and have been used widely as an animal model of human disease and in xenotransplantation research. In this study, we isolated, cultured, and identified pig SSCs *in vitro*, induced them to differentiate into adipocytes and neuron-like cells, and studied the expression of key genes. We hope that these results will further research on the use of SSCs in regenerative medicine.

## 2. Results

### 2.1. Identification of SSCs

**Figure 1:**
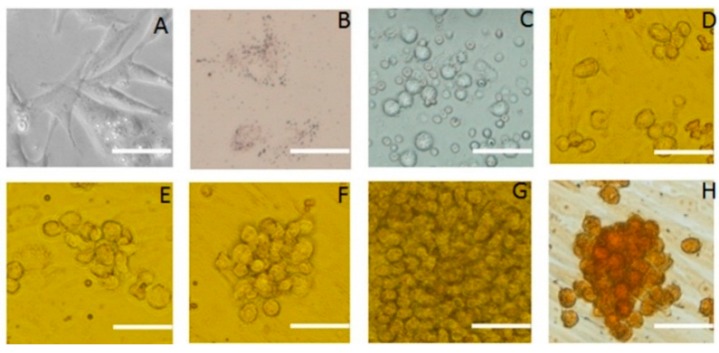
Morphology of SSCs and Sertoli cells in culture. Appearance under the microscope and various staining properties were used to identify the cells. (**A**) Sertoli cells derived from porcine testicular tissue; (**B**) Oil red-o staining of Sertoli cells; (**C**) Primary SSCs derived from porcine testicular tissue; (**D**–**G**) Colony of SSCs grown on feeder cells for 3, 5, 7, 12 days; (**H**) SSCs clone cells stained for alkaline phosphatase activity; the image indicates strong alkaline phosphatase activity. Scale bar: 5 μm.

SSCs and Sertoli cells were isolated from four to seven-day-old male Jiangquhui piglet testis by differential adhesion and SSCs were cultured on a feeder layer of Sertoli cells. The cultured cells were observed under the microscope. Typical images were shown in Figure 1. Sertoli cells showed as fibroblasts typically spread out and their nuclei had many nucleoli. Their cytoplasm had small bright vacuoles (Figure 1A). Four days after isolation, Sertoli cells began to proliferate quickly (Figure 1B). Staining with Oil red O revealed many red lipid droplets in the cytoplasm (Figure 1C). The SSCs were nearly spherical under the microscope and congregated around Sertoli cells. Within 48 h of culture, the SSCs were suspended in the medium (Figure 1D) and, after 11 days in culture, had typical grape-like morphology (Figure 1E). When stained for alkaline phosphatase, SSCs appeared brown and Sertoli cells appeared light or unstained (Figure 1F). The percentage of putative SSCs that were AKP-positive were 81.45%  ±  5.77%.

**Figure 2:**
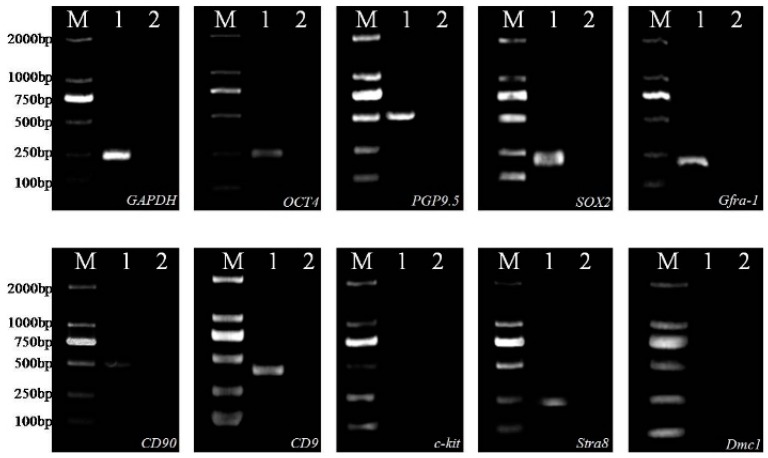
Expression of SSC-specific genes in cultured cells as determined by RT-PCR. M, DL2000 Marker used as a molecular size indicator; Lane 1, specific genes expression with cDNA of cultured cells; Lane 2, negative control with RNA of cultured cells; GAPDH used as positive control in cultured cells.

**Figure 3:**
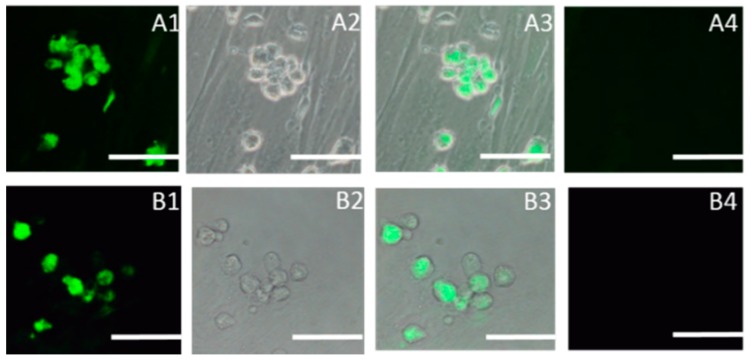
Immunocytochemical staining of SSCs using antibodies (FITC-conjugated secondary antibody, green) against SOX2 and SSEA-1 considered markers of pluripotency. (**A1**) SOX2, dark field; (**A2**) SOX2, bright field; (**A3**) Merged with dark field and bright; (**A4**) Negative control; (**B1**) SSEA-1, dark field; (**B2**) SSEA-1, bright field; (**B3**) Merged with dark field and bright; (**B4**) Negative control. Scale bar: 5 μm.

Spermatogonial stem cells cultured on Sertoli cells expressed the SSC marker genes *Oct4*, *PGP9.5*, *SOX2*, *Gfra-1*, *CD90*, *CD9*, and *Stra8*, but not *Dmc1* or *c-kit* (Figure 2). The expression of SOX2, SSEA1 (Stage-specific embryonic antigen-1), was detected in SSCs by indirect immunofluorescence (Figure 3).

### 2.2. Subculture of Pig SSCs

**Figure 4:**
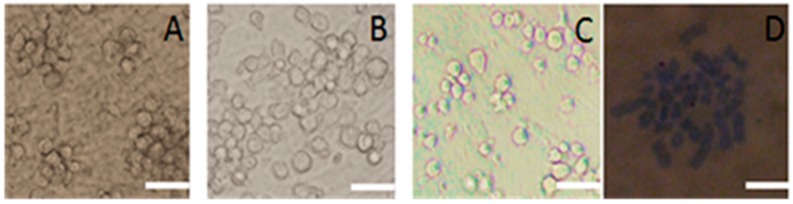
Images of cultured porcine SSCs after repeated subculture and their karyotype. (**A**) Cells in the fifth subculture; (**B**) Cells in the 10th subculture; (**C**) Cells in the15th subculture; (**D**) Karyotype cells in the 15th subculture. Scale bar: 5 μm.

In the first six passages of pig SSCs, the cells were round or oval and of similar size (Figure 4A). Their clones were also similar, but with the seventh passage of the cells, the SSCs size was no longer uniform, with cells that were both larger and smaller than in the previous subculture. By the tenth passage, cells were even less uniform and required about 15 days before the next subculture. The number of cells in the clones also decreased (Figure 4B). By the 15th passage, a cellular protuberance was apparent in the culture. This culture did not form clones until 18 days after subculture and there were fewer cells also in it (Figure 4C). Karyotype analysis confirmed that the chromosome numbers were 2*n* = 38, *n* = 19 (Figure 4D). In the next subculture, the cells were larger, approximately two to three times than the size of the previous subculture. Cells did not readily form clones or adhere, suggesting that they may have had the potential to differentiate.

### 2.3. Differentiation of SSCS into Neuron-Like Cells

**Figure 5:**
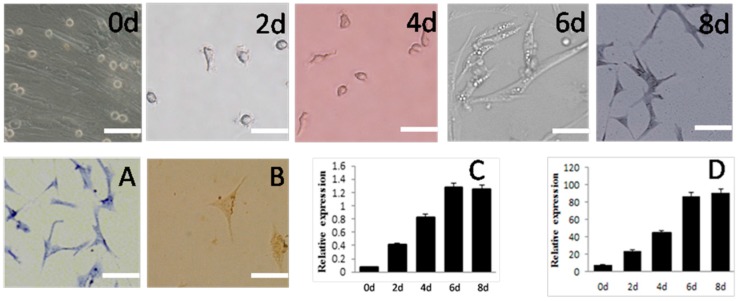
Morphology and gene expression of SSCs differentiated into neuron-like cells *in vitro*. Appearance under the microscope and various staining properties were used to confirm their identity.Schematic represents the strategy used for *in vitro* differentiation. 0d, 2d, 4d, 6d, 8d mean the same, 2th, 4th, 6th, 8th day after induction. (**A**) Toluidine blue staining of SSCs-derived cells; (**B**) Immunocytochemcal staining of SSCs-derived cells; (**C**) Expression of *Nestin* and (**D**) β*-tubulin*, both measured by mRNA levels during induction*.* Scale bar: 5 μm.

Third-passage SSCs were induced by 5.5 × 10^−5^ M β-Mercaptoethanol (β-ME), 1 × 10^−7^ M retinoic acid (RA), and 5 × 10^−4^ M 3-isobutyl-1-methylxanthine (IBMX). Beginning two days after transfer of cells to the induction medium, they began to form irregular outgrowths on the surface. On day four, the outgrowths began to gradually increase and the cells acquired different thicknesses and began to elongate, losing their spherical shape. By sox to eight days after induction, most of the SSCs resembled neurons (Figure 5). The cytoplasm was stained blue by toluidine blue (Figure 5A). These cells were identified as neurons with immunocytochemical staining using monoclonal antibodies against Neuron-Specific Enolase (NSE) (Figure 5B), which stains the cytoplasm darker than the nucleus. Expression of *Nestin* and β*-tubulin*, specific to neurons, increased during induction (Figure 5C,D) and was greatest six days after induction.

### 2.4. Differentiation of SSCs into Adipocytes

**Figure 6:**
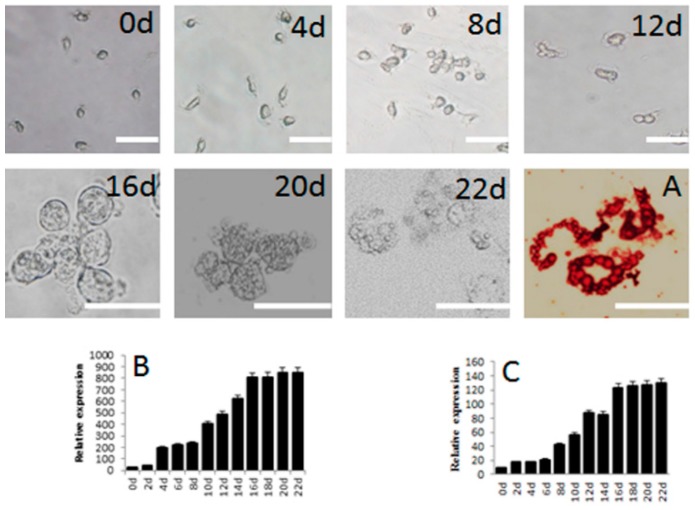
Morphology and gene expression of SSCs differentiated into adipocytes *in vitro*. Appearance under the microscope and various staining properties were used to confirm their identity. Schematic represents the strategy used for *in vitro* differentiation. 0d, 2d, 4d, 6d, 8d, 10d, 12d, 14d, 16d, 18d, 20d, 22d mean the same, 2th, 4th, 6th, 8th, 10th, 12th, 14th, 16th, 18th, 20th, 22th day after induction. (**A**) Oil red-O staining of adipocytes derived from SSCs; (**B**) Expression of *PPAR*γ and (**C**) *C/EBP*α, both measured by mRNA levels, during induction. Scale bar: 5 μm.

This process began with transfer of SSCs to the adipocyte induction medium A. The results are shown in Figure 6. There was no difference in cell morphology during the first 12 days of induction. By day 16 after induction, small lipid droplets began to appear in the cells, as apparent from the red color from staining with Oil red O (Figure 6A). The droplets gradually increased in size and dispersed from the nucleus. The expression of *PPAR*γ was detected 4 days after the start of induction, remained unchanged from day 4 to day 8, and increased from day 10 to day 16 (Figure 6B).

## 3. Discussion

Isolation and purification of SSCs are necessarily the first steps in any characterization of their biology and function. Several methods were used to isolate and purify SSCs, such as fluorescence-activated cell sorting (FACS), magnetic-activated cell sorting (MACS), differential plating, the selection with extracellular matrix (ECM), velocity sedimentation, or density gradient centrifugation(Reviewed by Zheng) [14]. Our approach was based on three well-established protocols. The two-step enzyme digestion is commonly employed for several reasons. The cell suspensions it yields are pure, single cells that have a high rate of survival and easily adhere to the culture flask. The differential plating method is also widely used in the purification of SSCs of domestic animals as the method is simple and the differences in the adherent rate of SSCs and other cells permits the isolation of large numbers of SSCs [34,35]. Finally, using Sertoli cells as the feeder layer provides a successful micro-environment for *in vitro* culture of SSCs [11,36]. In our study, collagenase and trypsin were used to digest the mesenchyme and the differential adherent method was used to purify the SSCs. The purity (90%) of gonocytes from neonatal pig testis are obtained via Nycodenz centrifugation followed by differential plating [7]. We can get SSCs with 81% of purity and we were able to subculture the SSCs *in vitro* to 15 generations of purification after the primary Sertoli cell isolation and culture.

SSCs specifically express several genes, notably *integrin α6*, *integrinβ1*, *CD9*, *CD24*, *SOX2*, and *Oct4*. In contrast, they also fail to express other genes: *Sca*, *c-kit*, *CD34*, *MHC-I*, and *CD51* [37,38,39,40,41,42]. For pigs, *PGP9.5*, *ZBTB16*, *CD90*, *GFRα1*, *NANOG2*, *POU5F1*, *SSEA1* have been proved as molecular markers of spermatogonia in testes [9,43,44,45,46,47,48]. These genes, then, are useful markers for identifying a cell culture as SSCs and were employed in this capacity here. We found the specific genes *OCT4*, *PGP9.5*, *SOX2*, *Gfra-1*, *CD90*, *CD9* expressed in the cells by RT-PCR. By indirect immunofluorescence, the expression of SOX2, SSEA1 was detected in SSCs. There were inherently fluorescent which can be found within the newborn pig testis interstitial cells [49]. In this study, we adjusted the microscope without any fluorescence to observe the control group, and then observed the experimental groups to assure there were no autofluorescence in visual field. The gene expression patterns that we observed support our conclusion that our cultured cells were SSCs. We believe that we have developed a method for the culture of highly pure SSCs that should establish the foundation for successful induction of differentiation of these cells. We next attempted to induce the differentiation of these SSCs into nervous cells and adipocytes.

Bone marrow stromal cells have been induced to differentiate into neuron-like cells as well as other cells. The inclusion of retinoic acid (RA), a vitamin A derivative, in the induction media increased the number of neuron cells and the formation of other neuroglia, including astroglial cells [50,51]. Thus, RA was included in the induction medium in our attempts to induce SSCs to differentiate into neuron-like cells. We found that RA, β-ME, and IBMX in the culture medium were necessary induce porcine SSCs to differentiate into neuron-like cells. We determined that the ideal concentrations were 1 µM RA and IBMX, and 10 µM β-ME. After two days in this medium, SSCs formed the irregular outgrowths typical of neurons.

We also assessed the NSE protein as well as expression of *Nestin* and β*-tubulin.* Nestin is an intermediate filament of the vertebrate-cell cytoskeleton and has been used to track the proliferation, migration, and differentiation of neuronal stem cells. *Nestin*, which is thought to be expressed exclusively and firstly in neural progenitor cells of the normal brain, is a neuron marker in adult rat and human brains [52] and, when detected in dental pulp stem cells (along with *Tub3*), was considered evidence for differentiation into neural cells. The expression of *Nestin* would decrease in adult nervous system [53]. The finding that *Nestin* did not decline at the end of induction in this study showed that the neuron-like cells we induced did not display properties that were consistent with the characteristics of normal neuron cells. β*-tubulin* is the main cytoskeleton protein of nerve cells and a protein marker for adult nerve cells [54,55]. NSE protein was detected in SSCs in the induction culture six days after transfer, as was expression of *Nestin* and β*-tubulin* based on the presence of their mRNA. The expression of β*-tubulin* was detected before the expression of *Nestin*, suggesting that microtubule formation was necessary or promontory to the differentiation of SSCs into neuron cells.

We next attempted to induce the SSCs to differentiate into adipocytes. In other cell types, insulin, IBMX, indometacin, and dexamethasone have been successfully used to induce differentiation into adipocytes [33]. In this study, we used two culture mediums that included insulin, IBMX, and dexamethasone for the induction of SSCs to adipocytes. Between 12 and 16 days after exposing the cultured cells into the induction medium, lipid droplets were present and fused continuously. In this study, we used Oil red O staining to identify Sertoli cells and adipocytes. Lipid droplets (LD) detected by Oil red O staining are cellular inclusion devoted to lipids storage. LD exist in Sertoli cells and adipocytes. This approach could also be considered as an effectively model to indentify Sertoli cells and adipocytes [56,57].

We also assessed the expression of two genes important in adipogenesis, *PPAR*γ and *C*/*EBP*α. *PPAR*γ is the key regulatory factor in the adipogenesis regulatory network and usually expressed earlier than most of the other genes believed to be involved in adipogenesis [58]. *PPAR*γ can regulate the expression of *C/EBP*α, lipid metabolism transporters, and cell secretory proteins to induce the differentiation of adipocytes [59]. In our study, we detected expression of *PPAR*γ earlier than we detected expression of *C/EBP*α in the cultured SSCs and the expression of *PPAR*γ remained low. The earlier expression of *PPAR*γ is consistent with its key regulatory role. *PPAR*γ is believed to regulate *C/EBPα* through an interaction between two proteins, not by binding the promoter of *C*/*EBP*α [60,61]. Interestingly, as a transcription factor in the terminal differentiation of adipocytes, *C*/*EBP*α also can regulate the expression of *PPAR*γ [62,63].

Cell signaling during stem cell differentiation is, no doubt, a complex process. Chemicals from other cells, contact with adjacent cells, and the chemical micro-environment can serve as signals that may affect differentiation. Is cell signaling the same *in vitro* and *in vivo*? If so, we can look into the mechanism of a particular cell type’s differentiation. In this study, we verified that porcine SSCs possess the capability to differentiate into neuron-like cells and adipocytes and that the process can be induced by chemical signals *in vitro*. The application of this information to tissue repair after injury is an area for future research, as is the question of how these cells could be transplanted into the animal body.

## 4. Experimental Section

### 4.1. Animal Material

Pig testes were acquired from four to seven-day-old male Jiangquhui boars when they were castrated on the Jiangsu Jiangquhai breeding pig farm. The animal-use protocol was approved by the laboratory-animal management and experimental-animal ethics committee of Yanzhou University.

### 4.2. Isolated and Cultured SSCs

#### 4.2.1. Preparation of Feeder Cells

Sertoli cells from the pig testes served as feeder cells and were isolated as described below according to the literature [64]. Testes were cut into pieces and digested with 1 mg/mL type IV collagenase (Sigma-Aldrich China, Inc. Shanghai, China) in Dulbecco’s modified Eagle medium (DMEM; Life Technologies Corporation, Shanghai, China) at 37 °C for 20 min, centrifuged at 1000 rpm for 5 min, and the pellet was collected. The pellet was digested with 0.25% Trypsin-EDTA (Life Technologies Corporation, Shanghai, China) until termination with 10% fetal bovine serum (FBS; Thermo Fisher HyClone, Shanghai, China) in DMEM. The digestion products were filtered through a 0.045 mm mesh nylon filter to remove large particles or cell clumps, centrifuged at 1000 rpm for 6–8 min, and the supernatant discarded. The pellet was re-suspended in DMEM with 10% FBS in DMEM and cultured at 37 °C and 5% CO_2_ for 2 h. The supernatants were pooled and put into another culture bottle at 37 °C and 5% CO_2_ for 2 h. The procedure was repeated twice more. The last time, the culture medium was discarded and new medium was added. The adherent cells were cultured at 37 °C and 5% CO_2_ until the culture was 80% confluent. The cells were treated with mitomycin C for 2 h and digested with 0.25% Trypsin-EDTA for 3 min, when it was terminated with 10% FBS in DMEM. The cells were seeded in 12-well plates at a density of 1 × 10^4^ cells/mL and cultured at 37 °C and 5% CO_2_ to prepare for culture of SSCs. Some of the cells were used for confirmation of their identity as Sertoli cells by Oil red O staining.

#### 4.2.2. Isolation and Culture of SSCs

SSCs were isolated from the feeder cells by the same procedure described above according to the literature [64]. After four series of differential attachment, the supernatant was centrifuged at 1000 rpm for 6–8 min. The pelleted cells were suspended in 10% FBS in DMEM supplemented 2% pig serum, 0.1 mM β-mercaptoethanol (β-ME), 2 mM l-glutamine, 1 µM sodium pyruvate, 1% non-essential amino acid, 0.1 ng/mL leukemia inhibitor factor (LIF), 5 ng/mL human stem cells factor (hSCF), 10 ng/mL basic fibroblast growth factor (bFGF, Sigma, Shanghai, China), and then cultured at 38 °C and 5% CO_2_ with pig Sertoli cells (as acquired above) as the feeder monolayer. After approximately 11 days in culture, the feeder cells typically formed a large number of colonies and were 100% confluent. Lastly, all cells were dissociated with 0.25% trypsin for 1 min and differential attachment was performed as above twice to isolate SSCs.

### 4.3. Identification of SSCs

#### 4.3.1. Staining for Alkaline Phosphatase Activity

The third-passage SSCs were identified by staining for alkaline phosphatase activity, which differentially stains stem cells [24]. The percentage of the positively stained cells was counted using a hemocytometer.

#### 4.3.2. Identification of SSC-Specific Gene Expression by Reverse Transcription-PCR (RT-PCR)

SSCs were further identified as such by identifying SSC-specific gene expression. Total RNA of the third-passage SSCs was extracted using Trizol reagent (Invitrogen, Life Technologies Corporation, Shanghai, China) according to the manufacturer’s instructions. Total RNA was treated with DNase I (Takara, Dalian, China) for 15 min at room temperature and incubated at 70 °C for 5 min before first-strand cDNA synthesis. Then the RNA was used to synthesize cDNA using the AM-MLV Reverse Transcriptase kit (Invitrogen)in a reaction volume of 50 µL. One microliter of first-strand cDNA was used for RT-PCR reactions. The primers which span at least one intron were designed by Primer3, according to related gene sequences from Genbank and the literature [65]. The sequences of the primers are shown in Table 1. PCR amplification was performed in a thermocycler (PTC-150 Minicycler PCR system; MJ Research Inc., Waltham, MA, USA) using rTaq polymerase (Takara) in a 25 μL reaction mixture consisting of an initial 5 min denaturation step at 95 °C, 35 cycles of 95 °C for 45 s, 55–58 °C for 30 s, 72 °C for 1 min, and a final extension for 10 min at 72 °C. Glyceraldehyde-3-phosphate dehydrogenase (GAPDH) gene was used as a positive control to confirm the presence of cDNA [66]. To conform that the RT-PCR signals derived not from genomic DNA, for each gene tested a negative control identical to the test assay using a RNA sample was included.

**Table 1:** Primers of SSC-specific genes and induced cell-specific genes.

Gene	Forward Primer (5′-3′)	Reverse Primer (5′-3′)	Product Size (bp)	Annealing Temperature (°C)
*OCT4*	GAGGAGTCCCAGGACATCAA	TCGTTGCGAATAGTAACTGC	248	56
*SOX2*	CAAGATGCACAACTCGGAGA	TGCTGTAGCTGCAGTTGCTC	236	58
*CD9*	TTCTGGCTCGCTGGGATT	CATCGGGAGGCTTGAGAGTA	468	55
*PGP9.5*	GAGATGCTGAACAAAGTGCTG	CATGGTTCACCGGAAAAGG	526	56
*CD90*	ACCATTGGCATCGCTCTCTT	GCCTTGTGGCTTCGTGTATCT	512	56
*Gfra-1*	GAACGGAGGCGGCAGACCAT	AAGCCCAGAGTAGGCGAGGAG	242	58
*Stra8*	ACCTCACAGCCTCAAAGTGG	CCTGGGGTTTCTGGAGTACA	248	57
*Dmc1*	TGGCTGTGTTTGTGACCAAT	CCCAATTCCTCCAGCAGTTA	323	55
*c-kit*	gatgccttcaaggatttgga	atggaatctgaggccttcct	181	56
*Nestin*	GGACAGTGAGGACAAGGCA	AACACGGGCTCTATCACCTC	281	57
*β-tubulin*	cttcccacgtctccacttct	tcttgttctgcacgttgagc	233	56
*PPARγ*	TACCAAAGTGCCATCAAA	TGGAGTGGAAATGCTGGA	110	56
*C/EBP-α*	cacttgcagttccagatcgc	taccgacttcttggctttgc	222	58
*GAPDH*	TTCCACGGCACAGTCAAGG	TCACGCCCATCACAAACATG	240	55

#### 4.3.3. Immunocytochemistry of SSC-Specific Genes

Third-passage SSCs were washed three times with phosphate-buffered saline (PBS) and fixed with 4% paraformaldehyde for 30 min. The cells were incubated with PBS supplemented with Tween20 (PBST) for 30 min and a specific antigen (SOX2, stage-specific embryonic antigen 1 (SSEA1; Yangzhou Qiuzhi Biotechnology Co., Yangzhou, China) overnight at −4 °C, while specific antigen was excluded in control group. After washing three times with PBST, fluorescein isothiocyanate (FITC)-labeled goat anti-mouse immunoglobulin M (Sigma) was added and the incubation was continued at 37 °C for 2 h. Cells were washed again with PBST and observed under an inverted fluorescence microscope(Olympus, Shinjuku, Japan).

#### 4.3.4. Subculture and Karyotype Analysis

When the SSC colonies had formed and feeder cells were 100% confluent, all cells were dissociated and subcultured. Karyotype analysis was based on Zhang [67].

### 4.4. Induced Differentiation of SSCs

Third-passage SSCs were differentially attached in 24-well plates covered by laminin. After two days, the medium was changed to induction media that should permit the differentiation of SSCs into neuron-like cells or adipocytes. The cells were observed and photographed every 24 h. The induced SSCs were identified by immunocytochemical staining and qRT-PCR for specific genes. qRT-PCR was performed using the 7500 System (ABI, Carlsbad, CA, USA) in a total volume of 20 μL containing SYBR mix (10 μL; Takara, Dalian, China), primers (4 ng), and cDNA sample (50 ng) according to the manufacturer’s instructions. Pig *GAPDH* was used as an internal reference to normalize relative gene expression. The level of gene expression was measured using the 2^−ΔΔ*C*t^ method. All PCR products were run on ethidium bromide-stained agarose gels and confirmed using melting curve analyses to assess product quality.

#### 4.4.1. Differentiation of SSCs into Neuron-like Cells

The induction medium was 90% DMEM, 10% FBS, 5.5 × 10^−5^ M β-ME, 1 × 10^−7^ M retinoic acid (RA), and 5 × 10^−4^ M 3-isobutyl-1-methylxanthine (IBMX) [50,68,69]. The induced SSCs were stained with toluidine blue and NSE. Expression of the neuron-specific *Nestin* and *β-tubulin* were identified by qRT-PCR every two days [70,71,72].

#### 4.4.2. Differentiation of SSCs into Adipocytes

One induction medium, referred to as medium A, was 90% DMEM, 10% FBS, 5.5 × 10^−5^ M β-ME, 1 µM hexadecadrol, 0.1 mg/L insulin, and 5 µM IBMX. A second induction medium, referred to as medium B, was 90% DMEM, 10% FBS, 5.5 × 10^−5^ M β-ME, and 0.1 mg/L insulin [73]. Induction medium A was added for three days and then replaced with induction medium B for one day. Next, the above procedures about using media A and media B were repeated three times. The induction medium B maintained the cultured cells for the duration of the experiment, when they were stained with Oil red O [24,68]. At this time, expression of the adipocyte-specific gene *peroxisome proliferator-activated receptor-γ* (*PPAR-γ*) and *C/EBPα* was detected by qRT-PCR [58].

### 4.5. Statistical Analyses

All data are given as the mean ± standard error. Statistical analyses were performed using least significant difference (LSD) tests with SPSS version 13.0 (IBM Corporation, Armonk, NY, USA).

## 5. Conclusions

In conclusion, using differential adherent method and Sertoli cells as the feeder layer, pig SSCs were able to be subcultured *in vitro* to 15 generations of purification. SSCs cultured *in vitro* specifically expressed several genes, CD9, CD90, SOX2, and SSEA1which were stem cells-specific genes. RA, β-ME, and IBMX in the culture medium induced porcine SSCs to differentiate into neuron-like cells. Expression of *Nestin* and β*-tubulin*, specific to neurons, could be detected after induction. Using two culture mediums including insulin, IBMX, and dexamethasone, pig SSCs could be induced to adipocytes on basis of emergence of lipid drops and expression of two important genes in adipogenesis, *PPAR*γ, and *C/EBP*α.
